# PREDICT‐PD: An online approach to prospectively identify risk indicators of Parkinson's disease

**DOI:** 10.1002/mds.26898

**Published:** 2017-01-16

**Authors:** Alastair J. Noyce, Lea R'Bibo, Luisa Peress, Jonathan P. Bestwick, Kerala L. Adams‐Carr, Niccolo E. Mencacci, Christopher H. Hawkes, Joseph M. Masters, Nicholas Wood, John Hardy, Gavin Giovannoni, Andrew J. Lees, Anette Schrag

**Affiliations:** ^1^ University College London Institute of NeurologyUniversity College LondonLondonUK; ^2^ Barts and the London School of Medicine and DentistryQueen Mary UniversityLondonUK; ^3^ Wolfson Institute of Preventative Medicine, Barts and the London School of Medicine and Dentistry, Queen Mary UniversityLondonUK; ^4^ Charing Cross HospitalImperial CollegeLondonUK; ^5^ Blizard Institute, Barts and the London School of Medicine and Dentistry, Queen Mary UniversityLondonUK

**Keywords:** Parkinson's disease, prodrome, cohort, epidemiology, risk factors

## Abstract

**Background:**

A number of early features can precede the diagnosis of Parkinson's disease (PD).

**Objective:**

To test an online, evidence‐based algorithm to identify risk indicators of PD in the UK population.

**Methods:**

Participants aged 60 to 80 years without PD completed an online survey and keyboard‐tapping task annually over 3 years, and underwent smell tests and genotyping for glucocerebrosidase (GBA) and leucine‐rich repeat kinase 2 (LRRK2) mutations. Risk scores were calculated based on the results of a systematic review of risk factors and early features of PD, and individuals were grouped into higher (above 15th centile), medium, and lower risk groups (below 85th centile). Previously defined indicators of increased risk of PD (“intermediate markers”), including smell loss, rapid eye movement–sleep behavior disorder, and finger‐tapping speed, and incident PD were used as outcomes. The correlation of risk scores with intermediate markers and movement of individuals between risk groups was assessed each year and prospectively. Exploratory Cox regression analyses with incident PD as the dependent variable were performed.

**Results:**

A total of 1323 participants were recruited at baseline and >79% completed assessments each year. Annual risk scores were correlated with intermediate markers of PD each year and baseline scores were correlated with intermediate markers during follow‐up (all *P* values < 0.001). Incident PD diagnoses during follow‐up were significantly associated with baseline risk score (hazard ratio = 4.39, *P* = .045). *GBA* variants or G2019S *LRRK2* mutations were found in 47 participants, and the predictive power for incident PD was improved by the addition of genetic variants to risk scores.

**Conclusions:**

The online PREDICT‐PD algorithm is a unique and simple method to identify indicators of PD risk. © 2017 The Authors. Movement Disorders published by Wiley Periodicals, Inc. on behalf of International Parkinson and Movement Disorder Society.

The prodromes of Parkinson's disease (PD) can begin many years before diagnosis^1^, ^2^ and offer an opportunity to identify individuals in the earlier stages of the disease.^3^ Studies aiming to identify patients at risk of PD have mainly centred on individuals with a family history of PD or asymptomatic carriers of genes associated with PD, idiopathic anosmia, rapid eye movement–sleep behavior disorder (RBD), or with imaging abnormalities associated with increased risk (such as hyperechogenicity on transcranial sonography and nigrostriatal deficit on dopamine transporter imaging).^4^, ^5^, ^6^, ^7^

These studies are limited by the rarity, representativeness, and/or feasibility of identifying these factors because of cost or availability and by the large numbers needing to be screened, given the incidence rate of PD of approximately 200 per 100,000 person‐years in those over the age of 60 years.^8^ Here we report 3‐year data on a novel method to recruit and identify individuals with risk indicators of PD through an online screening process using an algorithm derived from a meta‐analysis of known risk and prodromal features of PD.^9^

## Methods

### Data Collection

We previously reported the methods of a study to estimate and test risk of PD based on a systematic review of associations of risk factors and early PD features with a subsequent diagnosis of PD and prospective evaluation of a community‐based population.^9^, ^10^ In brief, participants were recruited via the study website following an advertising campaign in 2011 (which included emails to members of the Parkinson's UK charity). The study was approved by the Queen Square Research Ethics Committee (reference 10/H0716/85). Inclusion criteria at baseline included residency in the United Kingdom and age between 60 to 80 years. Exclusion criteria were preexisting and self‐reported PD, any other movement disorder, stroke, motor neuron disease, dementia, or drug usage known to be associated with iatrogenic parkinsonism.

The volunteers were prompted by email to return to the website and complete the tests on a yearly basis. The survey included demographic questions and items on early nonmotor features and risk factors for PD, including validated questionnaires, such as the Hospital Anxiety Depression Scale and the RBD Screening Questionnaire (RBDSQ) as well as questions that had been associated with increased risk of PD previously in observational studies.^9^, ^11^, ^12^ Participants undertook an online keyboard‐tapping task each year, the BRadykinesia Akinesia INcoordination test (BRAIN test; available at www.braintaptest.com), which has been validated to assess upper limb motor function in PD.^13^ The U.S. version of the University of Pennsylvania Smell Identification Test (UPSIT) was posted to them at baseline and in year 3, and the answers were completed both online and in the smell test booklets.^14^ Each year the survey included a question on whether the participants had been given any new diagnoses, and they were specifically asked about “Parkinson's disease” and/or “movement disorder.” Positive responses to these 2 diagnoses were followed up by telephone interview. For newly diagnosed patients with PD, the telephone interview was followed with a home visit and in‐person clinical examination by A.J.N. to ensure that U.K. Brain Bank diagnostic criteria were met and to gather further information.^15^ Participants lost to follow‐up were defined as those who did not complete submissions any year after baseline. No additional participants were recruited after the baseline year.

### Genotyping

Saliva collection tubes were dispatched alongside the smell tests to respondents of the year 3 follow‐up survey. Saliva was returned by post and DNA was extracted using standard methods. Direct Sanger sequencing was used to genotype participants for mutations in exons 8‐11 of glucocerebrosidase (GBA) (which contains >95% of known *GBA* pathogenic mutations) and exon 41 in the leucine‐rich repeat kinase 2 (LRRK2) gene was screened for the G2019S mutation.^16^ Further details of genetic analysis are provided in the supplementary material.

### Risk Score Ranking and Outcomes

Early features and risk factors assessed online were used to calculate a risk score (see supplementary material, including Supplementary Table 1), and participants were ranked according to their risk score. Because incident cases of PD were expected to be few during follow‐up, support for enrichment of the population at risk of PD was tested by examining associations between risk scores and the following previously established factors which are associated with a high risk of PD (intermediate markers): reduced sense of smell (assessed using the UPSIT), presence of subjective RBD (using the RBDSQ), and slowing of finger‐tapping speed (using the kinesia score [KS; number of alternate key taps in 30 seconds] for the slower hand). The Movement Disorder Society Task Force recently reported an increased risk of PD in those with hyposmia (likelihood ratio [LR] 4.0), subjective RBD (LR 2.3), and abnormal quantitative motor testing (LR 3.5).^17^ These 3 intermediate markers were therefore not included in the algorithm, but used as outcomes to assess the performance of the algorithm.

### Analyses

Annual risk estimates in all of the participants were ranked from highest to lowest, and this ranking was used to identify higher, middle, and lower risk groups. For this prospective analysis, we used centile‐based cut‐offs to determine higher (>15th centile) and lower (<85th centile) risk groups (rather than groups with fixed numbers) so that these groups would reduce in proportion to the overall number of participants during follow‐up.

A priori hypotheses for longitudinal follow‐up were that higher risk scores at baseline would be associated with abnormal scores on intermediate marker scales during follow‐up (longitudinal) and that higher risk scores each year would be associated with abnormal scores on intermediate marker scales (cross‐sectional). In an exploratory analysis, it was hypothesized that higher risk scores (with and without the inclusion of odds ratios associated with specific genetic variants), and intermediate markers would be associated with new diagnoses of PD.

### Statistical Methods

To assess reproducibility of the methods, risk scores were calculated annually. The associations between annual risk scores and intermediate markers that year were examined using regression, and higher and lower risk groups were compared for intermediate markers of PD that year (except smell, which was tested at baseline and year 3 only). Movement between higher, lower, and middle risk groups each year was also studied.

For longitudinal performance of the algorithm, the association of baseline risk scores with intermediate outcomes at follow‐up was examined, and baseline higher and lower risk groups were compared for intermediate markers during follow‐up. Descriptive information on individuals with a PD diagnosis during follow‐up was given, and an exploratory analysis of the association of baseline risk scores with later incident PD was performed.

KS (tapping speed) between groups was compared using *t* tests and described using means and 95% confidence intervals (CI). UPSIT and RBDSQ scores were not normally distributed, and medians and interquartile range and Wilcoxon rank sum tests were used. Comparisons for categorical data (defined by cut‐off values for each intermediate marker) were made using Fisher's exact test. Cut‐off values for smell loss, RBD, and tapping speed based on the 15th centile for each intermediate marker using UPSIT, RBDSQ, and KS were identified (scores of ≤27, ≥5, and ≤44, respectively). These were similar to those in the published literature.^12^, ^13^, ^18^ A chi‐square test for trend was used to compare the frequency of genetic variants between the higher, middle, and lower risk groups.

The relationships of risk scores in the entire dataset (independent variable) with UPSIT, RBDSQ, and KS (dependent variables) were examined using median, linear, and Poisson regression, respectively. Exploratory Cox regression was used to calculate hazard ratios (HR) for the association between baseline risk scores and incident PD and between baseline intermediate markers and incident PD. The regression model was repeated in the subset of participants for whom *GBA* and *LRRK2* status was known (with odds ratios associated with genetic risk factors included in the algorithm). Participants with newly diagnosed PD were excluded from the analyses of outcomes in the years following diagnosis. All analyses were performed using Stata (StataCorp LP, College Station, Texas).

## Results

**Figure 1:**
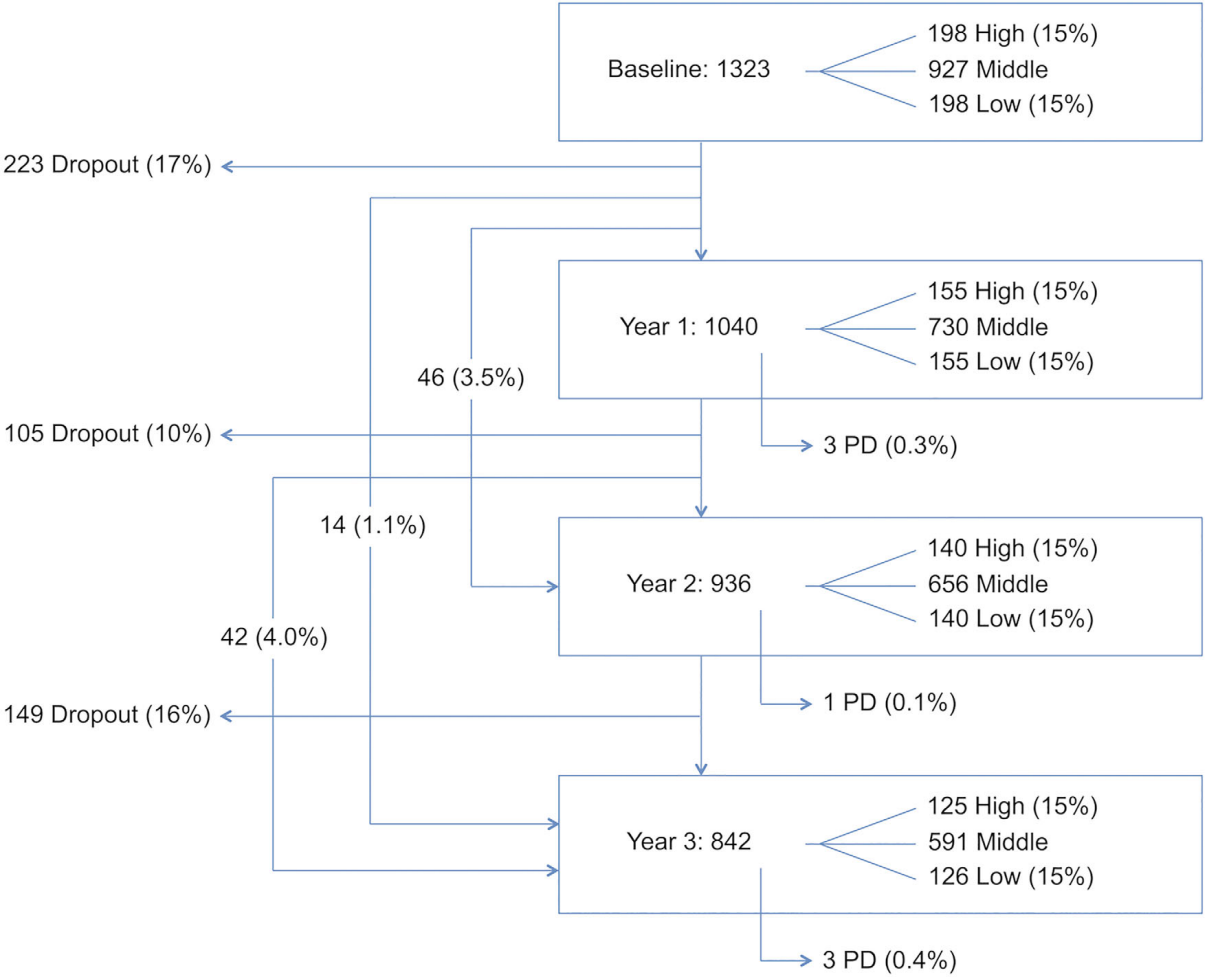
Participant numbers, dropout rates, and new diagnoses of PD. Note that some participants completed follow‐up assessments at year 2 but not year 1 (46 [3.5%]), at year 3 but not years 1 and 2 (14 [1.1%]), or at year 1 and 3 but not year 2 (42 [4.0%]).

At baseline, 1,323 eligible volunteers were recruited, and 1,040 of the voluteers completed follow‐up testing in year 1 (79% of baseline), 939 in year 2 (90% of year 1 and 71% of baseline respondents), and 846 in year 3 (90% of year 2 and 64% of baseline). A total of 223 participants (17%) completed the baseline assessment only. Using 15th centile risk cut‐offs, there were 198 participants each in the higher and lower risk groups at baseline, 155 in each group at year 1, 140 participants in each group in year 2, and 125 and 126 in the higher and lower risk groups in year 3, respectively (Fig. 1). Baseline data are presented in Table 1 and Supplementary Table 2. Demographic and risk factor data for each follow‐up year are presented in Supplementary Table 3. Those that continued to participate in the study were younger than those who were lost to follow‐up but were otherwise similar (Supplementary Table 4).

**Table 1:** Patient characteristics at baseline

	All	Higher risk	Lower risk
Age	66.2 (63.5‐70.5)	70.2 (67.1‐74.7)	63 (61.4‐64.6)
Female	806 (60.9%)	42 (21.2%)	170 (85.9%)
Current smoker	51 (3.9%)	3 (1.5%)	28 (14.1%)
Past smoker	541 (40.9%)	87 (43.9%)	88 (44.4%)
Coffee	1187 (89.7%)	173 (87.4%)	194 (98%)
Hypertension	348 (26.3%)	59 (29.8%)	75 (37.9%)
NSAID use	83 (6.3%)	6 (3%)	19 (9.6%)
CCB use	155 (11.7%)	30 (15.2%)	25 (12.6%)
Alcohol	1143 (86.4%)	179 (90.4%)	177 (89.4%)
1st degree relative	208 (15.7%)	74 (37.4%)	1 (0.5%)
Constipation	215 (16.3%)	73 (36.9%)	1 (0.5%)
Head injury	327 (24.7%)	86 (43.4%)	11 (5.6%)
Beta blocker use	103 (7.8%)	30 (15.2%)	15 (7.6%)
Depression/anxiety^a^	159 (12%)	37 (18.7%)	9 (4.5%)
Erectile dysfunction	180 (34.8%)	132 (84.6%)	0 (0%)

NSAID, nonsteroidal anti‐inflammatory drugs; CCB, calcium channel blockers.

a Hospital Anxiety Depression Scale score ≥ 11 (moderate); higher and lower risk are defined by the 15th and 85th centiles of risk scores, respectively.

### Longitudinal Analysis of Baseline Risk Scores With Outcomes Over 3 Years

Baseline risk scores were associated with significantly higher rates of all intermediate markers of PD during each year of follow‐up (*P* < 0.001; Supplementary Table 5). In addition, the higher risk group had significantly worse UPSIT, RBDSQ, and KS in all years than the lower risk group and had a greater proportion of individuals with smell loss, RBD, and slowed finger tapping according to predefined cut‐offs in every year of follow‐up (all *P* ≤ 0.003 except for the association between risk group and abnormal finger tapping in year 2 (*P* = 0.080; see Table 2 and Supplementary Table 6).

**Table 2:** Longitudinal associations of baseline risk scores with UPSIT, RBDSQ, and tapping speed at year 3

	Higher risk	Lower risk	*P* value^a^
UPSIT score			
n	130	132	
Median (IQR)	30 (26‐33)	33 (30‐35)	<.001
<27 (%)	40 (31)	15 (11)	<.001
RBDSQ score			
n	140	139	
Median (IQR)	2 (1‐4)	1 (0‐3)	<.001
>5 (%)	33 (24)	10 (7)	<.001
KS score			
n	135	130	
Mean (95% CI)	51.3 (49.5‐53.2)	55.5 (53.6‐57.4)	.001
<44 (%)	40 (30)	17 (13)	.002

IQR, interquartile range; CI,confidence interval; KS, kinesia score for the worst hand; RBDSQ, rapid eye movement sleep behavior disorder screening questionnaire; UPSIT, University of Pennsylvania smell identification test.

a *P* value from comparative analysis between higher and lower risk groups using Wilcoxon Rank Sum test for UPSIT and RBDSQ, *t* test for KS for continuous data, and Fisher's exact test for categorical data.

#### Comparison of Frequencies of Gene Variant Carriers by Baseline Risk

Sequencing of G2019S *LRRK2* mutation was successful for 806 samples (98% success). Comparisons with the reference sequence led to the detection of 2 (0.26%) heterozygous carriers of the G2019S *LRRK2* mutation.

Sequencing of *GBA* in 826 samples (of which 192 had been screened at an earlier stage^16^) yielded clear sequences for all screened exons in 800 participants (97% success). Comparisons with the reference sequence led to the detection of 45 carriers of *GBA* variant alleles of which 23 were E326K (20 heterozygous and 3 homozygous), 12 T369M (11 heterozygous and 1 homozygous), 8 N370S, 1 R463C, and 1 Rec*N*cil (recombinant allele associating L444P, A456P, and V460V). The overall frequency of *GBA* variants in the screened cohort was 5.45%.

The frequency of *GBA* carriers was 6.8% (10/146), 5.4% (28/516), and 5.0% (7/138) in the higher, middle, and lower risk groups, respectively. Comparing these frequencies using the chi‐square for trend did not provide evidence of association by risk group (*P* = 0.51).

### Cross‐Sectional Association of Risk Scores With Outcomes Each Year

Risk scores across the whole group were strongly associated with intermediate markers each year (all *P* values < 0.001). Higher and lower risk groups differed significantly in median UPSIT, RBDSQ, and mean KS score in all years of follow‐up (*P* values all ≤ 0.001; see Table 3 and Supplementary Table 7). In addition, the higher risk group had a greater proportion of individuals with smell loss, RBD, and slowed finger tapping according to predefined cut‐offs than the lower risk group each year (all *P* < 0.05).

**Table 3:** Cross‐sectional association of year 3 risk scores with UPSIT, RBDSQ, and tapping speed at year 3

	Higher risk	Lower risk	*P* value^a^
UPSIT score			
n	117	118	
Median (IQR)	30 (26‐33)	33 (30‐35)	<.001
<27 (%)	39 (33)	14 (12)	<.001
RBDSQ score			
n	125	126	
Median (IQR)	3 (1‐5)	1 (0‐3)	<.001
>5 (%)	34 (27)	5 (4)	<.001
KS score			
n	123	119	
Mean (95% CI)	50.9 (48.9‐52.9)	55.5 (53.4‐57.6)	.001
<44 (%)	36 (29)	19 (16)	.015

IQR, interquartile range; CI, confidence interval; KS, kinesia score for the worst hand; RBDSQ, rapid eye movement sleep behavior disorder screening questionnaire; UPSIT, University of Pennsylvania smell identification test.

a *P* value from comparative analysis between higher and lower risk groups using Wilcoxon Rank Sum test for UPSIT and RBDSQ, *t* test for KS for continuous data, and Fisher's exact test for categorical data.

#### Movement Between Groups

The majority of individuals remained in the same risk group (higher, middle, lower). However, annual changes of risk led to the movement of some participants between groups (Supplementary Figure). Between baseline and year 1, approximately 20% of both the higher and lower risk group moved to the middle risk group, in year 2, 26% and 15%, respectively, moved groups, and similar changes were observed in year 3. No participant moved from the higher to the lower risk group in any year, and 1 (0.1% of the cohort) from the lower to the higher risk group in year 1 only.

### Incident Diagnosis of PD During Follow‐Up

At year 1, 3 patients had been newly diagnosed with PD. Another participant was diagnosed in year 2, and 3 more in year 3. Of the participants with newly diagnosed PD at year 1, all 3 were in the higher risk group, and 2 had also been in the higher risk group at baseline. The participant diagnosed at year 2 was in the higher risk group at baseline, year 1, and year 2 year of follow‐up. Of the 3 participants diagnosed by year 3, all were in the middle risk group at baseline; 1 was in the higher risk group in the year prior to diagnosis, and there were marked increases in the rank of the risk estimates prior to diagnosis for the other 2 participants. On clinical examination, all patients satisfied the U.K. Brain Bank criteria for PD.^15^ Further details regarding rankings, intermediate markers, motor features, treatment, UPDRS motor scores, genetic information, and dopamine transporter imaging (where available) are provided in Supplementary Tables 8 and 9. There was substantial heterogeneity in the occurrence of intermediate markers in these individuals, with hyposmia in 4, reduced tapping speed in 5, and 4 reporting RBD symptoms according to predefined cut‐offs.

The incidence of independently diagnosed PD in subjects that had been in the higher risk group during 3 years of follow‐up was 1.6% per year (6 participants of 125 higher risk) and 0.2% across the whole cohort. Exploratory Cox regression analysis using incident PD over 3 years as the outcome showed an association with baseline risk estimate (HR 4.39; 95% CI 1.03‐18.68; *P* = 0.045). In the repeat analysis, restricted to participants for whom *GBA* and *LRRK2* status was known (789 participants), conservative odds ratios for the presence of variants associated with the risk of PD were included in the algorithm (see Supplementary Table 10). In this smaller sample, the association between baseline risk and incident PD was overall weaker (HR 3.44; 95% CI 0.85‐13.99; *P* = .084; see Table 4), but the addition of *GBA* and *LRRK2* variants in the algorithm improved the strength of association between baseline risk and incident PD (HR 4.22; 95% CI 1.21‐14.73; *P* = .024). Of the intermediate markers, baseline finger tapping was associated with incident PD at 3 years (HR 0.91; 95% CI 0.84‐0.98), but not RBDSQ. An association with baseline UPSIT scores could not be calculated because of small numbers (see Supplementary Table 8).

**Table 4:** Exploratory Cox regression analysis of association between baseline risk (with and without genetic variants), and baseline intermediate markers, with incident PD at 3 years of follow‐up

	HR (95% CI)	*P* value
Log risk without variants	3.44 (0.85‐13.99)	.084
Log risk with variants	4.22 (1.21‐14.73)	.024
UPSIT^a^	–	–
RBDSQ	1.21 (0.92‐1.59)	.177
KS	0.91 (0.84‐0.98)	.012

HR, hazard ratio; CI, confidence interval; KS, kinesia score for the worst hand; RBDSQ, rapid eye movement sleep behavior disorder screening questionnaire; UPSIT, University of Pennsylvania smell identification test.

a Baseline UPSIT data were only available on 3 of the 7 incident cases.

## Discussion

The online assessment of established risk factors and early features of PD has the potential to identify individuals with increased risk of PD from the general population. Our results suggest that the approach is effective and reproducible in identifying a group of individuals with increased risk markers of PD during a period of 3 years, and suggest enrichment of the population for incident PD. In this cohort, risk scores defined by the PREDICT‐PD algorithm at baseline and during each year of follow‐up were significantly associated with intermediate markers of PD (smell loss, RBD, and slowed tapping speed) at follow‐up. In addition, despite some movement between groups each year, the higher risk group had an increased rate of intermediate markers of PD during follow up, and higher baseline risk scores were associated with increased rate of incident PD. These results overall suggest that risk stratification using these methods is feasible. Although only a small number of individuals have been independently diagnosed with PD during follow‐up so far, the overall incidence of 0.2% is consistent with expected incidence rate in the age group of 60 to 80 years from the general population (1‐3 per 1000 per year^8^) and supports the representativeness of our sample. The higher risk group in this study was enriched approximately 5‐fold, supporting the overall findings of the study that the online algorithm can identify increased risk of PD in the population.

A total of 7 cases after a large population screening and follow‐up for 3 years is still a low number, but this is expected given the incidence of PD. However, this Internet‐based approach may be useful for population screening because it can easily be scaled upward. It will allow larger numbers of PD cases that represent the spectrum of the disease to be identified rather than what would be possible from cohorts of carriers of specific risk factors. It should be emphasized, however, that inclusion in the higher risk group alone is not predictive of subsequent PD diagnosis in individual participants.

Some movement was observed between higher, middle, and lower risk groups each year, as would be expected given the subjective nature of online surveying and the potential for symptoms to vary over time (eg, mood and bowel habit). Measures were taken to increase concordance through detailed instructions and personal communications to discount statistically and clinically improbable data (see supplementary material), but some inaccuracy may have been introduced through these methods. However, this would be more likely to increase the noise and reduce the significance of findings rather than lead to differential misclassification. The maximum percentage change within a group was seen in the higher risk participants between years 1 and 2 (26% moving to the middle risk group), but in general changes were limited to less than 20%, and no patient in the higher risk group switched to the lower risk group. Overall, stratification may therefore be most informative if repeated.

The inclusion of genetic variants in the algorithm improved the prediction of incident PD over the risk algorithm alone. The frequency of *GBA* variants was 5.45%, which is slightly higher than that of healthy controls (4.24%) in other studies^19^; but a frequency of 0.26% was found for the G2019S *LRRK2* mutation, similar to previous observations.^20^ The distribution of mutation carriers across the baseline risk groups showed a slight gradient in direction of the higher risk group, but it was not statistically significant and was in contrast to that observed in preliminary subgroup testing.^16^ The previously observed finding may have resulted from undersampling in the middle risk group, giving rise to low statistical power and the potential for false positives. However, when G*BA* and *LRRK2* effects were included in the model with the risk algorithm, the strength of association increased between baseline risk and incident PD at 3 years of follow‐up. Of the baseline intermediate markers of PD, only finger tapping at baseline was associated with incident PD at 3 years. This is likely because subjective RBD symptoms only occurred in 2 of the 7 incident cases, and UPSIT scores from baseline were only available for 3 of the 7 incident cases. Overall the number of converters currently is too small to draw robust conclusions, but these follow‐up results are encouraging.

The clustering of early nonmotor features has been studied in the PARS (Parkinson Associated Risk Study) and Tübinger evaluation of Risk factors for the Early detection of NeuroDegeneration (TREND) studies, but the co‐occurrence of features still requires further clarification. In PARS, participants with hyposmia were more likely to report altered mood (anxiety and depression), RBD symptoms, and constipation.^18^ In those who went on to have dopamine transporter imaging, hyposmia, male gender, and constipation combined effectively to predict dopaminergic deficit.^5^ In the TREND study, which recruited based on the presence of 1 or more of depression, RBD, and anosmia, the clustering of prodromal features was also observed.^21^

Cohorts that follow high‐risk groups, such as those with genetic mutations or RBD, provide important insights into the long‐term prodrome of PD, may provide platforms for biomarker studies and treatment trials, and guide research in this area. However, the strategies that measure multiple prodromal features and generate composite exposure information will be more likely to yield the greatest sensitivity and specificity for future diagnosis than individual markers. A recent development in this area is the new Criteria for Prodromal Parkinson's Disease from the MDS Task Force.^17^ When compared with the aforementioned studies, PREDICT‐PD has recruited participants with a much wider spectrum of risk for PD than those recruiting based on selected prodromal markers. Such wider recruitment might offer a better representation of the spectrum of PD, and acknowledge that not all that go on to be diagnosed with PD may not have those specific markers.

### Limitations

We acknowledge the selection bias that may have occurred in the recruitment of participants, including a mailshot through Parkinson's UK, the resulting high frequency of participants with a family history of PD, and therefore a small excess in the prevalence of *GBA* variants. However, we did not use family history as an entry criterion, and the proportion with a positive family history was lower than in other landmark studies such as TREND and PARS.^5^, ^21^ There was also an overall drop out of 17% of participants who only completed the baseline assessment but no follow‐up assessments. This may also have introduced selection bias, although there were no differences between those with or without follow‐up, meaning that bias as a result of loss to follow‐up was less likely. Notably, the greatest loss to follow‐up was between baseline and year 1 follow‐up because the study was funded to run as a cross‐sectional study before further funding enabled the cohort to be followed over time. Information relating to a new diagnosis of PD relied on self‐report and the retention of participants within the study. Reporting bias or differential loss to follow‐up could therefore have played a role in the observed results, but it is reassuring that there were no obvious differences between the participants who continued to participate and those who dropped‐out, reducing the probability of such bias. Although two intermediate markers were measured objectively, a potential limitation to our approach was the use a subjective questionnaire for one intermediate marker, the RBDSQ.^22^

The identification of only 7 individuals over 3 years with the current algorithm is a limitation for implementation. However, the ease of application makes enlargement of the cohort and validation in other cohorts a possibility. Future work is required to refine the algorithm, identify individual components with the greatest contribution, and reduce movement between groups. Combining the current algorithm with a more extensive genetic risk scoring approach may significantly improve predictive capabilities.^23^ Longer follow‐up with in‐depth study (including imaging and eventual postmortem examination) will identify a greater number of participants who have developed PD to allow further characterization of the higher and lower risk groups and refinement of the algorithm. Finally, our results will need to be replicated in independent samples in different populations.

## Author Roles

1) Research project: A. Conception, B. Organization, C. Execution; 2) Statistical Analysis: A. Design, B. Execution, C. Review and Critique; 3) Manuscript: A. Writing of the first draft, B. Review and Critique

A.J.N.: 1A, 1B, 2C, 3A

J.P.B.: 1A, 1B, 2C, 3A, 3B

L.P.: 2B, 3A, 3B

K.A.‐C.: 2B, 3A, 3B

L.R'B.: 2B, 3A, 3B

N.E.M.: 2B, 3A, 3B

C.H.H.: 1A, 1B, 2C, 3A, 3B

J.M.M.: 1A, 1B, 2C, 3A, 3B

N.W.: 1A, 1B, 2C, 3A, 3B

J.H.: 1A, 1B, 2C, 3A, 3B

G.G.: 1A, 1B, 2C, 3A, 3B

A.J.L.: 1A, 1B, 2C, 3A, 3B

A.S.: 1A, 1B, 2C, 3A

## Financial Disclosures of all authors (for the preceding 12 months)

A.J.N. reports salaries from Parkinson's UK and Barts Health National Health Service Trust; grants from Parkinson's UK, Élan/Prothena Pharmaceuticals, and GE Healthcare; shares from LifeLab Ltd.; advisory board member at myHealthPal; and honoraria Office Octopus, Henry Stewart Talks, and Britannia Pharmaceuticals Ltd.

N.E.M. reports a salary from the Department of Health's National Institute for Health Research (NIHR) Biomedical Research Centres funding streams.

G.G. has received compensation for serving as a consultant or speaker for, or has received research support from AbbVie, Bayer Schering Healthcare, Biogen Idec, Canbex, Eisai, Elan, Five Prime Therapeutics, Genzyme, Genentech, GlaxoSmithKline, Ironwood Pharmaceuticals, Merck‐Serono, Novartis, Pfizer, Roche, Sanofi‐Aventis, Synthon BV, Teva Pharmaceutical Industries, and Vertex Pharmaceuticals. A.J.L. reports grants from the Franes and Renee Hock Fund; consultancy fees from Britannia Pharmaceuticals (Genus) and BIAL Portela; and honoraria from Teva, Lundbeck, BIAL, Roche, Britannia, UCB, and Nordiclnfu Care.

A.S. reports grants from Economic and Social Research Council, GE Healthcare, Parkinson's UK; consultancy fees from Novartis, Boehringer Ingelheim, Astra Zeneca, Merck, Osmotica, AMGEN, and ACADIA Pharmaceuticals; and shares of Astra Zeneca. J.P.B., L.P., K.A.‐C., L.R'B., C.H.H., J.M.M., N.W., and J.H. report no disclosures.

## Supporting information

Additional Supporting Information may be found in the online version of this article at the publisher's website.

Supplementary Information
