# Supplementary material for: PREDICT‐PD: An online approach to prospectively identify risk indicators of Parkinson's disease
Source: Mov Disord. 2017 Jan 16;32(2):219–26. doi: 10.1002/mds.26898 (PMC5324558; doi:10.1002/mds.26898)
Supplement: Supplementary file 1 — Supplementary Information [file MDS-32-219-s001.docx]

**SUPPLEMENTARY DATA**

**Table 1: Effect size estimates for factors included in the risk algorithm derived from meta-analysis.[9,10]**

|  | Factor OR/RR |
| --- | --- |
| Age | See equation in main text derived from reference 8 |
| Female | 0.67 |
| Current smoker | 0.44 |
| Past smoker | 0.78 |
| Coffee | 0.67 |
| Hypertension | 0.74 |
| NSAID use | 0.83 |
| CCB use | 0.90 |
| Alcohol | 0.90 |
| 1st degree relative | 3.23 |
| Constipation | 2.34 |
| Head injury | 1.58 |
| Beta blocker use | 1.28 |
| Depression/anxiety | 1.86 |
| Erectile dysfunction | 3.80 |

Legend: NSAID = non-steroidal anti-inflammatory drugs; CCB = calcium channel blockers; OR=odds ratio; RR=relative risk

**Table 2: Intermediate marker analysis at baseline, showing associations between risk score and markers in the whole group, and comparative analysis between higher and lower risk groups.**

|  | All | p-value* | Higher risk | Lower risk | p-value** |
| --- | --- | --- | --- | --- | --- |
| UPSIT score |  |  |  |  |  |
| n | 888 |  | 138 | 130 |  |
| Median | 32 | <0.001 | 31 | 33 | <0.001 |
| IQR | 29 - 34 |  | 28 - 34 | 31 - 35 |  |
| <27 (%) | 135 (15%) |  | 30 (22%) | 7 (5%) | <0.001 |
|  |  |  |  |  |  |
| RBDSQ score |  |  |  |  |  |
| n | 1323 |  | 198 | 198 |  |
| Median | 2 | <0.001 | 2.5 | 2 | <0.001 |
| IQR | 1 - 3 |  | 1 - 4 | 0 - 3 |  |
| >5 (%) | 202 (15%) |  | 46 (23%) | 23 (12%) | 0.003 |
|  |  |  |  |  |  |
| KS score |  |  |  |  |  |
| n | 1079 |  | 161 | 161 |  |
| Mean | 53.6 | <0.001 | 50.9 | 54.6 | 0.002 |
| CI | 53 - 54.2 |  | 49.3 - 52.4 | 52.9 - 56.3 |  |
| <44 (%) | 181 (17%) |  | 34 (21%) | 27 (17%) | 0.394 |

IQR = interquartile range; CI = confidence interval; KS = kinesia score for the worst hand; RBDSQ = REM sleep behaviour disorder screening questionnaire; UPSIT = University of Pennsylvania smell identification test. * p-value derived from regression analysis of continuous measure of intermediate marker on continuous risk estimate (median regression used for association with UPSIT, Poisson regression for association with RBDSQ and linear regression for association with KS). ** p-value from comparative analysis between higher and lower risk groups using Wilcoxon Rank Sum test for UPSIT and RBDSQ, t-test for KS for continuous data, and Fisher’s exact test for categorical data.

**Table 3: Demographic information in each follow-up year, divided according to risk group.**

|  |  | Year 1 | | | |  | Year 2 | | | |  | Year 3 | | | |
| --- | --- | --- | --- | --- | --- | --- | --- | --- | --- | --- | --- | --- | --- | --- | --- |
|  |  | All | Higher risk | Middle risk | Lower risk |  | All | Higher risk | Middle risk | Lower risk |  | All | Higher risk | Middle risk | Lower risk |
| Age |  | 67.4 (64.9-71.3) | 71.2 (68.5-74.9) | 67.6 (65.1-71.2) | 64.5 (62.7-65.8) |  | 68.1 (65.7-72) | 71.6 (68.4-76.3) | 68.4 (66-72) | 65.5 (63.8-67.5) |  | 69.1 (66.7-72.9) | 72.6 (69.7-76.3) | 69.1 (66.9-72.6) | 66.7 (64.9-68.5) |
| Female |  | 642 (61.9%) | 34 (21.9%) | 474 (65.2%) | 134 (86.5%) |  | 582 (62.2%) | 27 (19.3%) | 427 (65.2%) | 128 (91.4%) |  | 512 (60.8%) | 22 (17.6%) | 373 (63.4%) | 115 (91.3%) |
| Current smoker |  | 29 (2.8%) | 1 (.6%) | 13 (1.8%) | 15 (9.7%) |  | 22 (2.4%) | 1 (.7%) | 9 (1.4%) | 12 (8.6%) |  | 20 (2.4%) | 2 (1.6%) | 6 (1%) | 12 (9.5%) |
| Past smoker |  | 416 (40.1%) | 71 (45.8%) | 283 (38.9%) | 62 (40%) |  | 371 (39.7%) | 65 (46.4%) | 255 (38.9%) | 51 (36.4%) |  | 334 (39.7%) | 56 (44.8%) | 232 (39.5%) | 46 (36.5%) |
| Coffee |  | 945 (91.1%) | 142 (91.6%) | 652 (89.7%) | 151 (97.4%) |  | 869 (92.9%) | 131 (93.6%) | 598 (91.3%) | 140 (100%) |  | 791 (93.9%) | 116 (92.8%) | 546 (92.9%) | 126 (100%) |
| Hypertension |  | 278 (26.8%) | 43 (27.7%) | 175 (24.1%) | 60 (38.7%) |  | 266 (28.4%) | 45 (32.1%) | 164 (25%) | 57 (40.7%) |  | 244 (29%) | 35 (28%) | 160 (27.2%) | 49 (38.9%) |
| NSAID use |  | 56 (5.4%) | 8 (5.2%) | 40 (5.5%) | 8 (5.2%) |  | 49 (5.2%) | 7 (5%) | 33 (5%) | 9 (6.4%) |  | 42 (5%) | 6 (4.8%) | 29 (4.9%) | 7 (5.6%) |
| CCB use |  | 121 (11.7%) | 25 (16.1%) | 70 (9.6%) | 26 (16.8%) |  | 119 (12.7%) | 21 (15%) | 69 (10.5%) | 29 (20.7%) |  | 135 (16%) | 25 (20%) | 83 (14.1%) | 26 (20.6%) |
| Alcohol |  | 915 (88.2%) | 139 (89.7%) | 633 (87.1%) | 143 (92.3%) |  | 836 (89.4%) | 126 (90%) | 578 (88.2%) | 132 (94.3%) |  | 756 (89.8%) | 109 (87.2%) | 523 (88.9%) | 121 (96%) |
| PD in 1st degree relative |  | 179 (17.3%) | 63 (40.6%) | 116 (16%) | 0 (0%) |  | 173 (18.5%) | 65 (46.4%) | 108 (16.5%) | 0 (0%) |  | 155 (18.4%) | 56 (44.8%) | 98 (16.7%) | 0 (0%) |
| Constipation |  | 185 (17.9%) | 55 (35.5%) | 129 (17.8%) | 1 (.6%) |  | 155 (16.6%) | 52 (37.1%) | 103 (15.7%) | 0 (0%) |  | 137 (16.3%) | 35 (28%) | 100 (17%) | 1 (.8%) |
| Head injury |  | 320 (30.9%) | 77 (49.7%) | 233 (32%) | 10 (6.5%) |  | 335 (35.8%) | 71 (50.7%) | 251 (38.3%) | 13 (9.3%) |  | 317 (37.6%) | 72 (57.6%) | 232 (39.5%) | 11 (8.7%) |
| Beta blocker use |  | 92 (8.9%) | 24 (15.5%) | 58 (8%) | 10 (6.5%) |  | 79 (8.4%) | 26 (18.6%) | 47 (7.2%) | 6 (4.3%) |  | 77 (9.1%) | 25 (20%) | 47 (8%) | 5 (4%) |
| Depression/anxiety* |  | 131 (12.6%) | 30 (19.4%) | 96 (13.2%) | 5 (3.2%) |  | 122 (13%) | 31 (22.1%) | 87 (13.3%) | 4 (2.9%) |  | 108 (12.8%) | 31 (24.8%) | 72 (12.2%) | 4 (3.2%) |
| Erectile dysfunction |  | 145 (36.7%) | 107 (88.4%) | 38 (15%) | 0 (0%) |  | 143 (40.5%) | 96 (85%) | 47 (20.6%) | 0 (0%) |  | 154 (46.7%) | 93 (90.3%) | 60 (27.9%) | 0 (0%) |
| *HADS score ≥ moderate; NSAID = non-steroidal anti-inflammatory drugs; CCB = calcium channel blockers | | | | | | | | | | | | | | | |

**Table 4: Demographic information and outcome variables at baseline in subjects that remained under follow-up and those lost to follow-up**

|  | Followed up | Lost to follow-up | p-value* |
| --- | --- | --- | --- |
| n | 1100 | 223 |  |
| Median age (IQR) | 66.1 (63.5 to 69.9) | 67.5 (63.5 to 72.3) | 0.015 |
| Female | 672 (61.1%) | 134 (60.1%) | 0.780 |
| Median UPSIT (IQR) | 32 (30 to 34) | 32 (28 to 35) | 0.589 |
| Median RBDSQ (IQR) | 2 (1 to 3) | 2 (1 to 4) | 0.480 |
| Mean KS (95% CI) | 53.9 (53.2 to 54.5) | 52.2 (50.5 to 53.9) | 0.065 |

IQR = interquartile range; CI = confidence interval; KS = kinesia score for the worst hand; RBDSQ = REM sleep behaviour disorder screening questionnaire; UPSIT = University of Pennsylvania smell identification test. *p-value derived from Wilcoxon Rank Sum test for non-parametric data and unpaired t-test for parametric data, and Fisher’s exact test for categorical data.

**Table 5: Regression analysis in the whole cohort demonstrating associations between baseline risk scores and intermediate markers each year.**

|  | Year 1 | | Year 2 | | Year 3 | |
| --- | --- | --- | --- | --- | --- | --- |
|  | All | p-value* | All | p-value* | All | p-value* |
| UPSIT score |  |  |  |  |  |  |
| n |  |  |  |  | 783 |  |
| Median (IQR) | N/A | | N/A | | 32 (29 – 34) | <0.001 |
| <27 (%) |  |  |  |  | 128 (16%) |  |
|  |  |  |  |  |  |  |
| RBDSQ score |  |  |  |  |  |  |
| n | 1034 |  | 935 |  | 839 |  |
| Median (IQR) | 2 (1 – 3) | <0.001 | 2 (1 – 3) | <0.001 | 2 (1 – 3) | <0.001 |
| >5 (%) | 125 (12%) |  | 128 (14%) |  | 103 (12%) |  |
|  |  |  |  |  |  |  |
| KS score |  |  |  |  |  |  |
| n | 878 |  | 792 |  | 791 |  |
| Mean (95% CI) | 54.1 (53.4 - 54.8) | <0.001 | 54.2 (53.4 - 54.9) | <0.001 | 53.8 (53.0 - 54.6) | <0.001 |
| <44 (%) | 149 (17%) |  | 132 (17%) |  | 160 (20%) |  |

IQR = interquartile range; CI = confidence interval; KS = kinesia score for the worst hand; RBDSQ = REM sleep behaviour disorder screening questionnaire; UPSIT = University of Pennsylvania smell identification test. * p-value derived from regression analysis of continuous measure of intermediate marker on continuous risk estimate (median regression used for association with UPSIT, Poisson regression for association with RBDSQ and linear regression for association with KS).

**Table 6: Associations of baseline risk scores with year 1 and 2 UPSIT, RBDSQ and KS scores.**

|  | Year 1 | | | Year 2 | | |
| --- | --- | --- | --- | --- | --- | --- |
|  | Higher risk | Lower risk | p-value** | Higher risk | Lower risk | p-value** |
| UPSIT score |  |  |  |  |  |  |
| n |  |  |  |  |  |  |
| Median (IQR) | N/A | | | N/A | | |
| <27 (%) |  |  |  |  |  |  |
|  |  |  |  |  |  |  |
| RBDSQ score |  |  |  |  |  |  |
| n | 153 | 158 |  | 147 | 152 |  |
| Median (IQR) | 2 (1 - 4) | 1 (0 - 3) | <0.001 | 2 (1 - 4) | 1 (0 - 3) | <0.001 |
| >5 (%) | 34 (22%) | 13 (8%) | 0.001 | 34 (23%) | 10 (7%) | <0.001 |
|  |  |  |  |  |  |  |
| KS score |  |  |  |  |  |  |
| n | 125 | 131 |  | 130 | 126 |  |
| Mean (95% CI) | 51.7 (49.7 - 53.7) | 57.3 (55.5 - 59.1) | <0.001 | 51.6 (49.9 - 53.3) | 55.7 (53.7 - 57.6) | 0.001 |
| <44 (%) | 31 (25%) | 13 (10%) | 0.003 | 30 (23%) | 18 (14%) | 0.080 |

IQR = interquartile range; CI = confidence interval; KS = kinesia score for the worst hand; RBDSQ = REM sleep behaviour disorder screening questionnaire; UPSIT = University of Pennsylvania smell identification test. ** p-value from comparative analysis between higher and lower risk groups using Wilcoxon Rank Sum test for UPSIT and RBDSQ, t-test for KS for continuous data, and Fisher’s exact test for categorical data. Note year three data is provided in the main body of the manuscript.

**Table 7: Association of risk estimates at year 1 and 2 with UPSIT, RBDSQ and KS scores.**

|  | Year 1 | | | Year 2 | | |
| --- | --- | --- | --- | --- | --- | --- |
|  | Higher risk | Lower risk | p-value** | Higher risk | Lower risk | p-value** |
| UPSIT score |  |  |  |  |  |  |
| n |  |  |  |  |  |  |
| Median (IQR) | N/A | | | N/A | | |
| <27 (%) |  |  |  |  |  |  |
|  |  |  |  |  |  |  |
| RBDSQ score |  |  |  |  |  |  |
| n | 154 | 154 |  | 140 | 140 |  |
| Median (IQR) | 2 (1 - 4) | 1 (0 - 3) | <0.001 | 2 (1 - 5) | 1 (1 - 3) | <0.001 |
| >5 (%) | 30 (19%) | 13 (8%) | 0.008 | 35 (25%) | 7 (5%) | <0.001 |
|  |  |  |  |  |  |  |
| KS score |  |  |  |  |  |  |
| n | 131 | 128 |  | 123 | 114 |  |
| Mean (95% CI) | 52.2 (50.1 - 54.3) | 57.6 (55.7 - 59.6) | <0.001 | 51.8 (49.9 - 53.6) | 56.2 (54.3 - 58.2) | 0.001 |
| <44 (%) | 37 (28%) | 15 (12%) | 0.001 | 29 (24%) | 14 (12%) | 0.028 |

Legend: IQR = interquartile range; CI = confidence interval; KS = kinesia score for the worst hand; RBDSQ = REM sleep behaviour disorder screening questionnaire; UPSIT = University of Pennsylvania smell identification test. ** p-value from comparative analysis between higher and lower risk groups using Wilcoxon Rank Sum test for UPSIT and RBDSQ, t-test for KS for continuous data, and Fisher’s exact test for categorical data. Note year three data is provided in the main body of the manuscript.

**Table 8: Annual rankings, risks and intermediate marker scores in seven subjects diagnosed with PD during follow-up.**

| **Subject** | **Risk score rank (high to low)** | | | | **Risk expressed as log odds** | | | | **UPSIT score** | | **RBDSQ score** | | | | **KS score** | | | |
| --- | --- | --- | --- | --- | --- | --- | --- | --- | --- | --- | --- | --- | --- | --- | --- | --- | --- | --- |
|  | **yr0** | **yr1** | **yr2** | **yr3** | **yr0** | **yr1** | **yr2** | **yr3** | **yr0** | **yr3** | **yr0** | **yr1** | **yr2** | **yr3** | **yr0** | **yr1** | **yr2** | **yr3** |
| A | **86** | **68*** | **67** | 253 | 13.6 | 12.1 | 11.7 | 31.7 | NA | 20 | 0 | 1 | 3 | 1 | 24 | 33 | 39 | 22 |
| B | **106** | **96*** | **115** | **45** | 15.9 | 14.7 | 16.3 | 8.3 | 35 | 35 | 12 | 13 | 12 | 12 | 56 | 60 | 55 | NA |
| C | 884 | **64*** | **28** | **60** | 114.6 | 11.7 | 5.9 | 10.4 | 29 | 34 | 2 | 2 | 6 | 6 | 57 | 54 | NA | 72 |
| D | **19** | **13** | **2*** | **2** | 5.2 | 4.8 | 2.4 | 2.3 | 24 | 22 | 7 | 9 | 4 | 7 | 41 | 38 | 40 | 37 |
| E | 1086 | NA | 233 | 219* | 155.1 | NA | 29.5 | 27.1 | NA | 14 | 2 | NA | 4 | 3 | 43 | NA | 42 | 29 |
| F | 319 | **52** | 173 | 165* | 38.6 | 9.6 | 21.1 | 5.3 | NA | 25 | 0 | 0 | 0 | 0 | 41 | 43 | 26 | 23 |
| G | 475 | **155** | NA | **57*** | 58.0 | 20.3 | NA | 9.8 | NA | 31 | 1 | 3 | NA | 5 | NA | 86 | NA | 38 |

Legend: yr = year; NA = not available; UPSIT = University of Pennsylvania smell identification test; RBDSQ = REM sleep behaviour disorder screening questionnaire; KS = kinesia score. *denotes the year that PD diagnosis was reported in the survey. Please note that the ranking is a relative score compared to other participants. Rankings in bold indicate that participant would have been in the higher risk group (>15^th^ centile) of risk estimates. Risk is given as the log odds of PD, with a ratio of 1:x. Lower values of x indicate indicate higher risk of PD.

**Table 9: Further information relating to the clinical diagnosis of Parkinson’s disease in 7 incident cases.**

| **Participant** | **Diagnosed** | **Presenting features** | **Diagnosis by** | **Medication** | **L-dopa response** | **Imaging** | **Date of visit** | **Motor UPDRS** | **GBA/LRRK2 mutation** |
| --- | --- | --- | --- | --- | --- | --- | --- | --- | --- |
| A | 2011 | Tremor left arm, rigidity, bradykinesia, smell loss ~5 years earlier | Neurologist | Sinemet | Good | N/A | 18/11/12 | 26 | Negative |
| B | 2011 | Tremor right arm, reduced arm swing, rigidity, bradykinesia, depression | Neurologist | Stalevo | Good | N/A | 07/09/15 | 24 | Negative |
| C | 2012 | Tremor, rigidity, bradykinesia | Neurologist | Madopar | Moderate | N/A | 28/04/14 | 9 | Negative |
| D | 2014 | Tremor left arm, rigidity, bradykinesia, restless legs syndrome | Neurologist | Ropinirole | Good | Abnormal DaTSCAN | 04/04/14 | 31 | Negative |
| E | 2014 | Stooped posture and hesitant gait. MSA suspected initially, revised to PD* | Neurologist | Madopar | Moderate | Abnormal DaTSCAN | 16/04/14 | 8 | Negative |
| F | 2014 | Tremor right arm, bradykinesia, difficulty with fine motor, depression | Neurologist | Sinemet | Good | N/A | 15/09/15 | 26 | GBA (RecNcil) |
| G | 2014 | Rigidity, bradykinesia, stooped posture | Neurologist | Madopar | Good | Abnormal DaTSCAN | 07/09/15 | 38 | Negative |

Legend: *note symptom onset 2012

**Table 10: Odds ratios for *LRRK2* and *GBA* variants from published literature along with conservative ORs used for modeling within the algorithm.**

|  | Odds ratio | 95% confidence interval | Conservative OR |
| --- | --- | --- | --- |
| *LRRK2* |  |  |  |
| - G2019S[24] | 9.6 | 6.4-14.4 | 9 |
|  |  |  |  |
| *GBA* |  |  |  |
| - RecNcil[25] | 7.3 | 1.7-66.4 | 7 |
| - R463C | NA | NA | 7 |
| - N370S[26] | 3.5 | 2.6-4.8 | 3.5 |
| - E326K[27] | 1.7 | 1.0-2.7 | 1.7 |
| - T369M[27] | 2.5 | 1.5-4.3 | 1.7 |

Legend: Odds ratios for variants come from a variety of studies in different populations, with different confounding structures and biases. G2019S, RecNcil and R463C are rare in controls and are strongly associated with PD. For R463C there is no estimated level of effect from case-control studies but it appears to have similar pathogenicity as RecNcil. For these three variants a conservation OR of 7–9 was estimated. N370S is a milder variant that is well described (OR=3.5), and E326K and T369M are milder still and a conservative OR was estimated as being 1.7 for each.

**SUPPLEMENTARY METHODS**

**Construction of the algorithm**

The preliminary algorithm was based on results of studies that provided a relative risk or odds ratio, as reported previously.[10] For each individual, the age-related risk (expressed as an odds) of developing PD was determined using an equation based on results from the Physicians Health Study.[8]

Odds of PD = 1 : 28.53049 + 73.67057e^(-0.165308(age-60))^

As these data were from an all-male cohort, and prevalence of PD is approximately 1.5 times greater in men, the age-related odds for women was reduced accordingly.[28] An individual’s risk was increased or decreased by each additional risk factor according to the strength of association with PD reported in the systematic review (see supplementary table 1). For example a male, current smoker with a 1:100 age-related odds of developing PD was calculated to have an odds of 0.44x1:100=1:227. If that individual also had a family history of PD their odds was calculated as 4.45x1:227=1:51. The odds for all participants were ranked and the 15% with the highest risk and 15% with the lowest risk scores were identified.

Exposures were ascertained in a binary fashion, except for age, which was included as a continuous variable. Where data existed on multiple levels (i.e. bowel movement frequency, erectile function, anxiety and depression), the same cut-offs were used as at baseline (see supplementary material). Laxative and antidepressant use were included as surrogate markers to suggest constipation and depression respectively. Information on use of medications for erectile dysfunction (ED) was only collected in year three.

**Coding of categorical data**

Based on the results of systematic review, which provided risk estimates for each early non-motor feature or risk factor significantly associated with altered risk of PD, a preliminary algorithm was developed to provide PD risk estimates for each participant. The algorithm included age, gender, smoking status, first degree relative with PD, coffee use, alcohol use, hypertension, NSAID use, calcium channel blocker use, beta blocker use, constipation, previous head injury, anxiety or depression and erectile dysfunction (in males only). Most factors were sought in binary terms (i.e. presence or absence) except for bowel movement frequency (7 possible answers for frequency with a cut off of less than 1 movement per day denoting low frequency or laxative use), erectile dysfunction (3 options with ‘poor’ indicating dysfunction) and mood (a cut off score of 11 or above in either the anxiety or depression components of the HADS questionnaire denoting moderate forms of these disorders or antidepressant use). In order to keep the survey simple, pesticide exposures, proxies for organo-chemical exposure, and more complicated factors were not included.

**Handling inconsistencies in data**

Steps to eliminate inconsistencies in the reporting of risk factors and early non-motor features were employed, such as checking that those that reported ED were male. A standardized method for handling inconsistencies was used, in which newly reported presence of factors such as head injury or family history were accepted as plausible, as well as being a smoker one year and ex-smoker the following year. However, where a factor had been reported previously and in a subsequent year not reported, data from all years were reviewed to determine whether the factor should be included or excluded. For example, in a subject that reported a past head injury at baseline and at year 1 and 3, but not at year 2, the year 2 data were re-coded so that the subject would be recorded as having a past head injury for all years of follow-up. Where the presence of a factor had been reported in 50% or more years of follow-up, it was re-coded as being present for all years. If reported in less than 50% it was re-coded as having been absent. These methods were applied to head injury, and smoking, coffee and alcohol consumption, and family history, where further information was not available (see table 11).

**Table 11: Proportion of subjects that had changes made to reported risk factors to improve consistency**

| **Factor** | **Number (%) amended for consistency in year 3** |
| --- | --- |
| Smoking | 149 (17.6%) |
| Coffee | 56 (6.6%) |
| Alcohol | 72 (8.5%) |
| Head injury | 132 (15.6%) |

**Quality control of intermediate markers**

BRAIN-tap test results were excluded from the analysis if KS was implausibly low (<20 taps in 30 seconds) or high (>110 taps in 30 seconds), since this suggested that the test instructions had been misunderstood or, that the test was performed using two hands together rather than separately. Other cases were excluded if their dysmetria score (DS) was greater than or equal to 1.5, indicating at least half the keystrokes were to the wrong key. These cut-offs were assigned based on previous BRAIN-tap test data (see reference).

Answers to smell tests were reported online and in the hard copies of the UPSIT booklet. Comparison was made between online answers and hard copies, with online answers chosen in preference to the hard copy answers (where both were available). Where there were discrepancies of 5 or more answers, the test result was excluded from the analysis.

**Molecular genetic analysis**

Participants provided saliva samples in specialized collection tubes returned via postal mail. DNA was extracted using standard methods. The fragment spanning exons 8-11 of *GBA* was amplified using PCR conditions and primers previously described.[29] Each reaction was performed in a 15µL volume containing 7.5µL of FastStart PCR master mix (Roche), 5µL of water, 0.5µL of each primer (5pmol/L), and 30 ng of genomic DNA. Clean-up of the PCR product was performed using ExoSAP-IT (Affymetrix Inc.). Sanger sequencing was used to screen exons 8, 9 and 10-11 separately using primers previously designed.[30] Exon 41 of the LRRK2 gene was amplified using primers and PCR conditions available on request. Sequencing reactions were performed in both forward and reverse directions for each exon of the *GBA* fragment and exon 41 of *LRRK2* using BigDye Terminator v3.1 sequencing chemistry and loaded on the ABI3730xl genetic analyzer (Applied Biosystems, Foster City, CA). These sequences were compared to the RefSeq reference (NM_000157.3 for *GBA* and NM_198578.3 for *LRRK2*) using Sequencher software (version 4.9; Gene Codes). Confirmation of the presence of each mutation was ascertained by resequencing. Nomenclature for all *GBA* mutated alleles refer to the mature protein i.e. not including the 39-residue signal peptide. Nomenclature for the *LRRK2* mutation, i.e. G2019S, refers to the mature protein sequence.

**Figure - Changes to risk groups during follow-up and new diagnoses of PD.**

Legend: H = higher risk group, M = middle risk group, and L = lower risk group. X = non-completion for that year. *Note that 1 new diagnosis of PD at year 3 did not complete the assessments at year 2 and therefore is not labelled on this figure.*


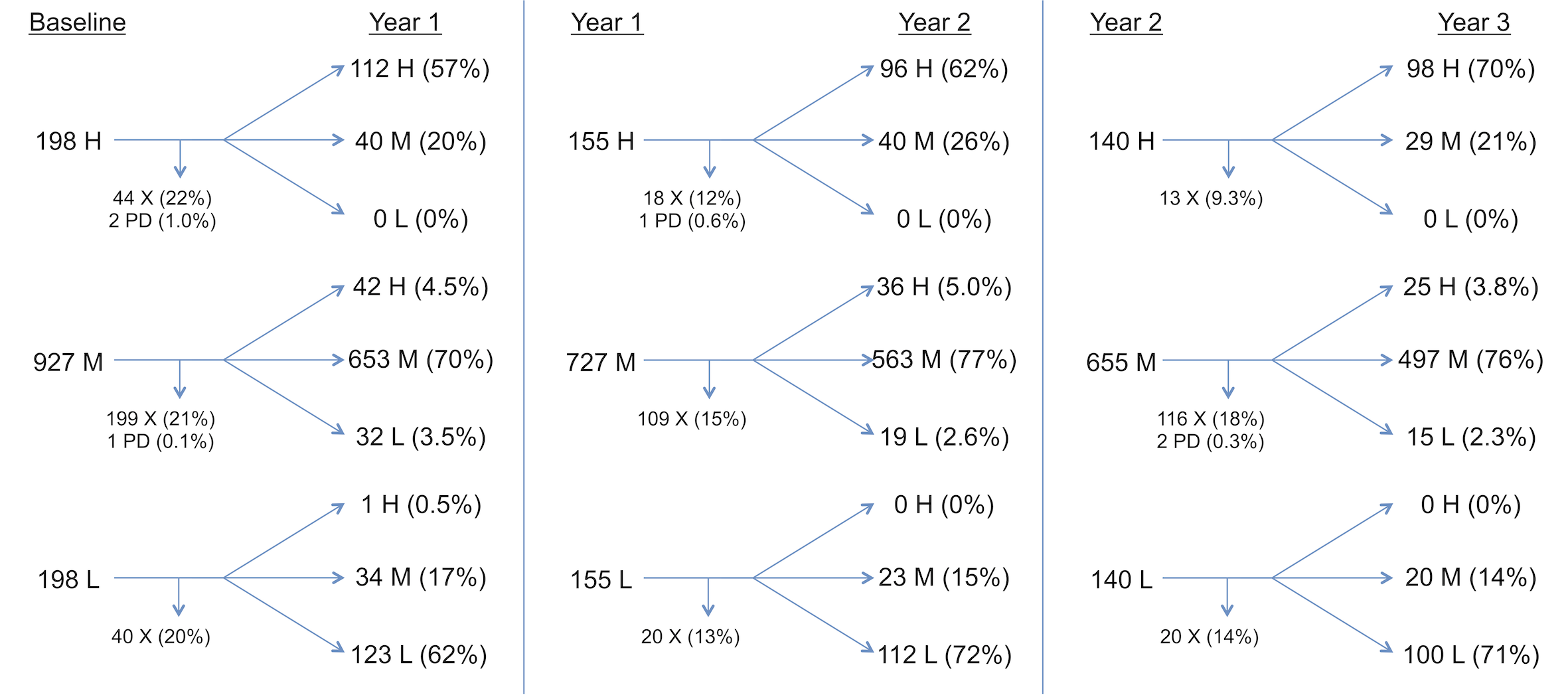


**SUPPLEMENTARY MATERIAL REFERENCES**

24 Do CB, Tung JY, Dorfman E, et al. Web-based genome-wide association study identifies two novel loci and a substantial genetic component for Parkinson’s disease. PLoS Genetics 2011;7(6):e1002141

25 Mitsui J, Mizuta I, Toyoda A, et al. Mutations for Gaucher disease confer high susceptibility to Parkinson disease. Arch Neurol 2009;66(5):571-576.

26 Lill CM, Roehr JT, McQueen MB, et al. Comprehensive research synopsis and systematic meta-analysis in Parkinson’s disease genetics: the PDGene database. PLoS Genetics 2012;8(3):e1002548

27 Benitez BA, Davis AA, Jin SC, et al. Resequencing analysis of five Mendelian genes and the top genes from genome-wide association studies in Parkinson’s disease. Molecular Neurodegeneration 2016 April 19^th^ [Epub ahead of print].

28 Wooten GF, Currie LJ, Bovbjerg VE, et al. Are men at greater risk for Parkinson's disease than women? J Neurol Neurosurg Psychiatry 2004;75:637–639.

29 Stone DL, Tayebi N, Orvisky E, et al. Glucocerebrosidase gene mutations in patients with type 2 Gaucher disease. Hum Mutat 2000;15(2):181-188

30 Neumann J, Bras J, Deas E, et al. Glucocerebrosidase mutations in clinical and pathologically proven Parkinson’s disease. Brain 2009;132:1783-1794.
